# Correction: Field assessment of the operating procedures of a semi-quantitative G6PD Biosensor to improve repeatability of routine testing

**DOI:** 10.1371/journal.pone.0300016

**Published:** 2024-02-29

**Authors:** The PLOS ONE Staff

## Notice of republication

This article was republished on February 21, 2024, to correct errors in the figures. Figs 1, 2, 6, and 7 were incorrectly published. The publisher apologizes for the error. Please download this article again to view the correct version. The originally published, uncorrected article and the republished, corrected articles are provided here for reference.

## Supporting information

S1 File Originally published, uncorrected article. (PDF)

S2 File Republished, corrected article. (PDF)
