# Supplementary figures and images for: Field assessment of the operating procedures of a semi-quantitative G6PD Biosensor to improve repeatability of routine testing
Source: PLoS One. 2024 Jan 19;19(1):e0296708. doi: 10.1371/journal.pone.0296708 (PMC10798449; doi:10.1371/journal.pone.0296708)

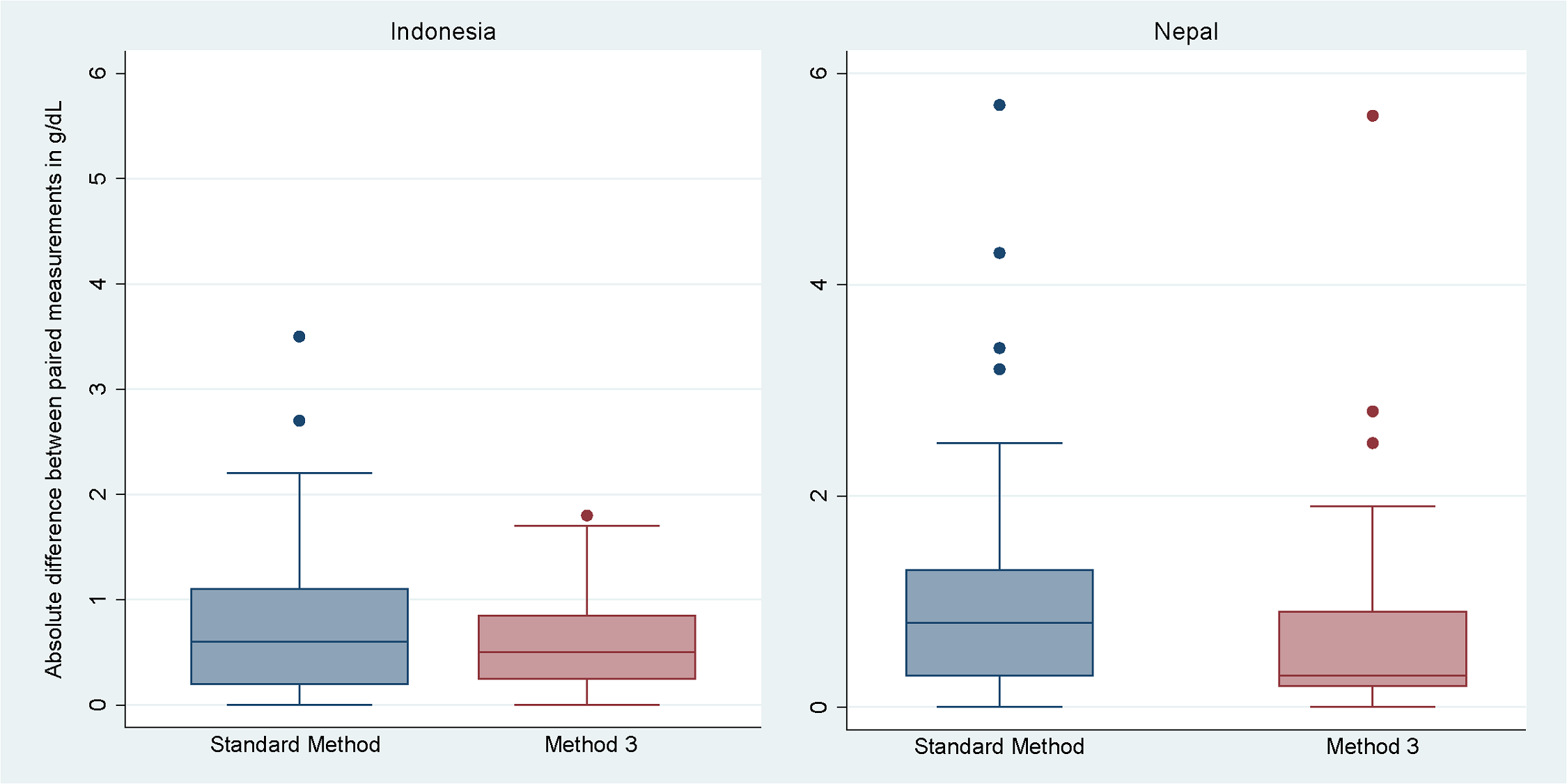

Supplement: S1 Fig — Boxplot of absolute difference per paired Biosensor Hb readings per method from field studies in Indonesia (left) and Nepal (right). (TIF) [file pone.0296708.s001.tif]

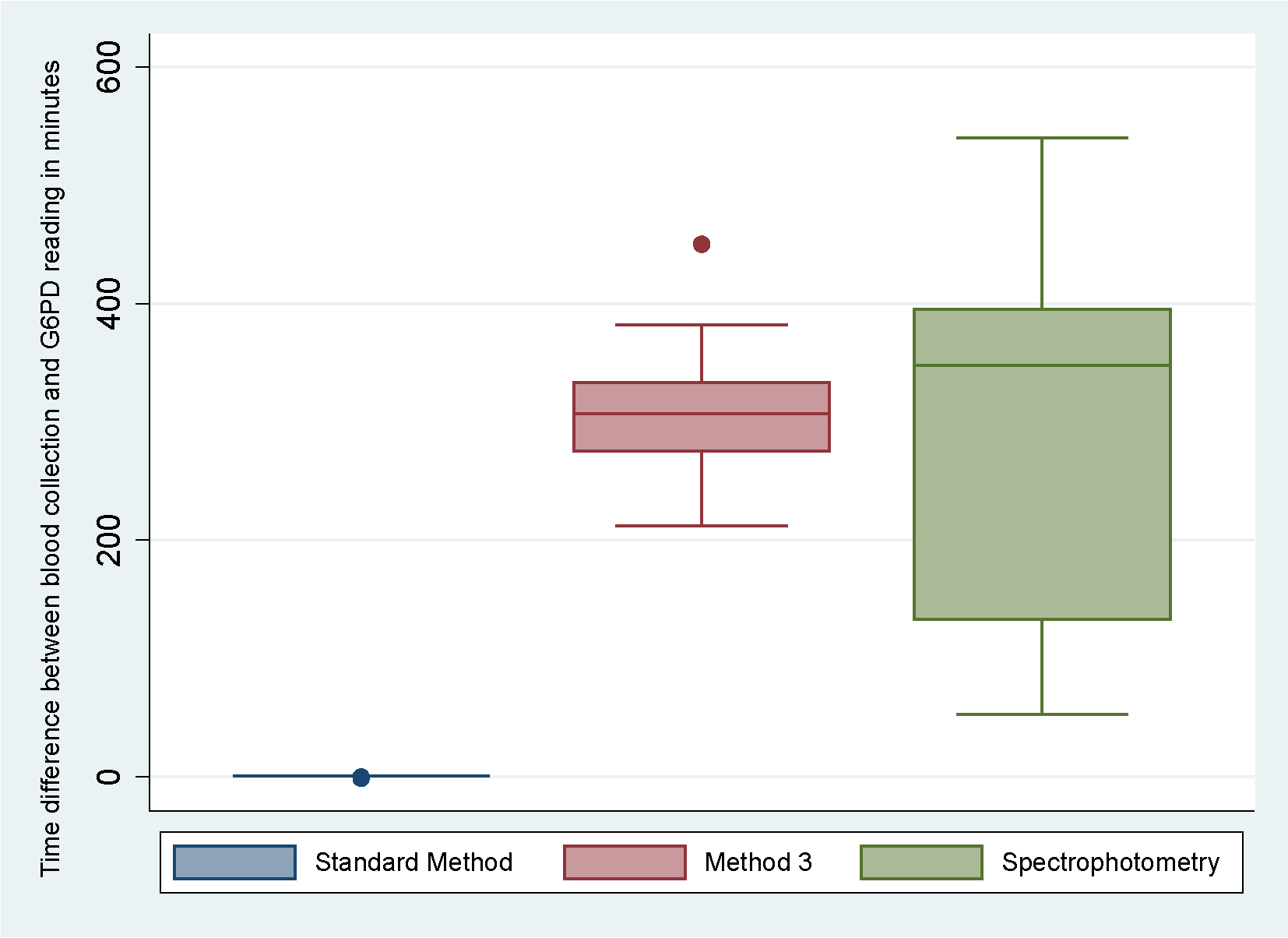

Supplement: S2 Fig — (TIF) [file pone.0296708.s002.tif]

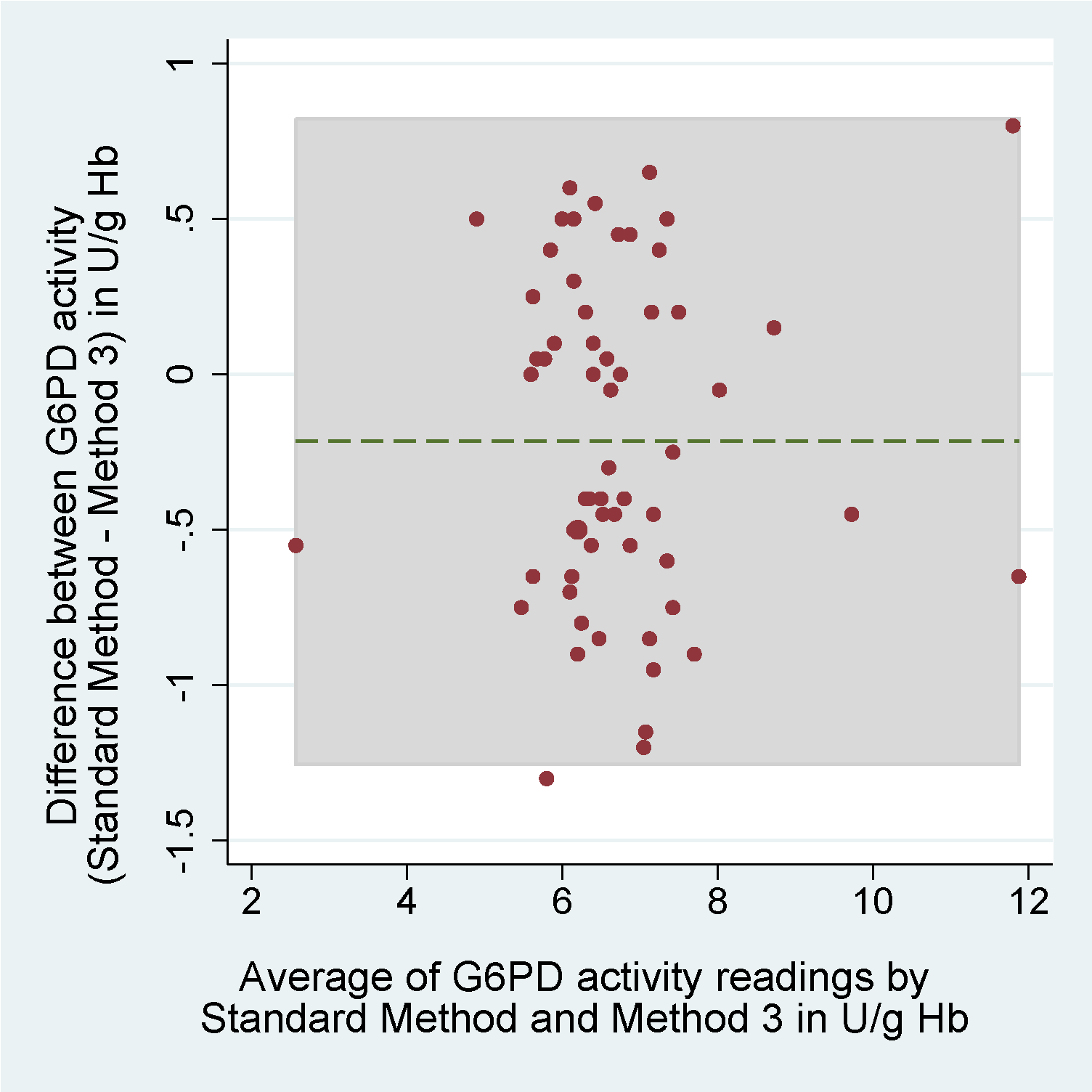

Supplement: S3 Fig — (TIF) [file pone.0296708.s003.tif]

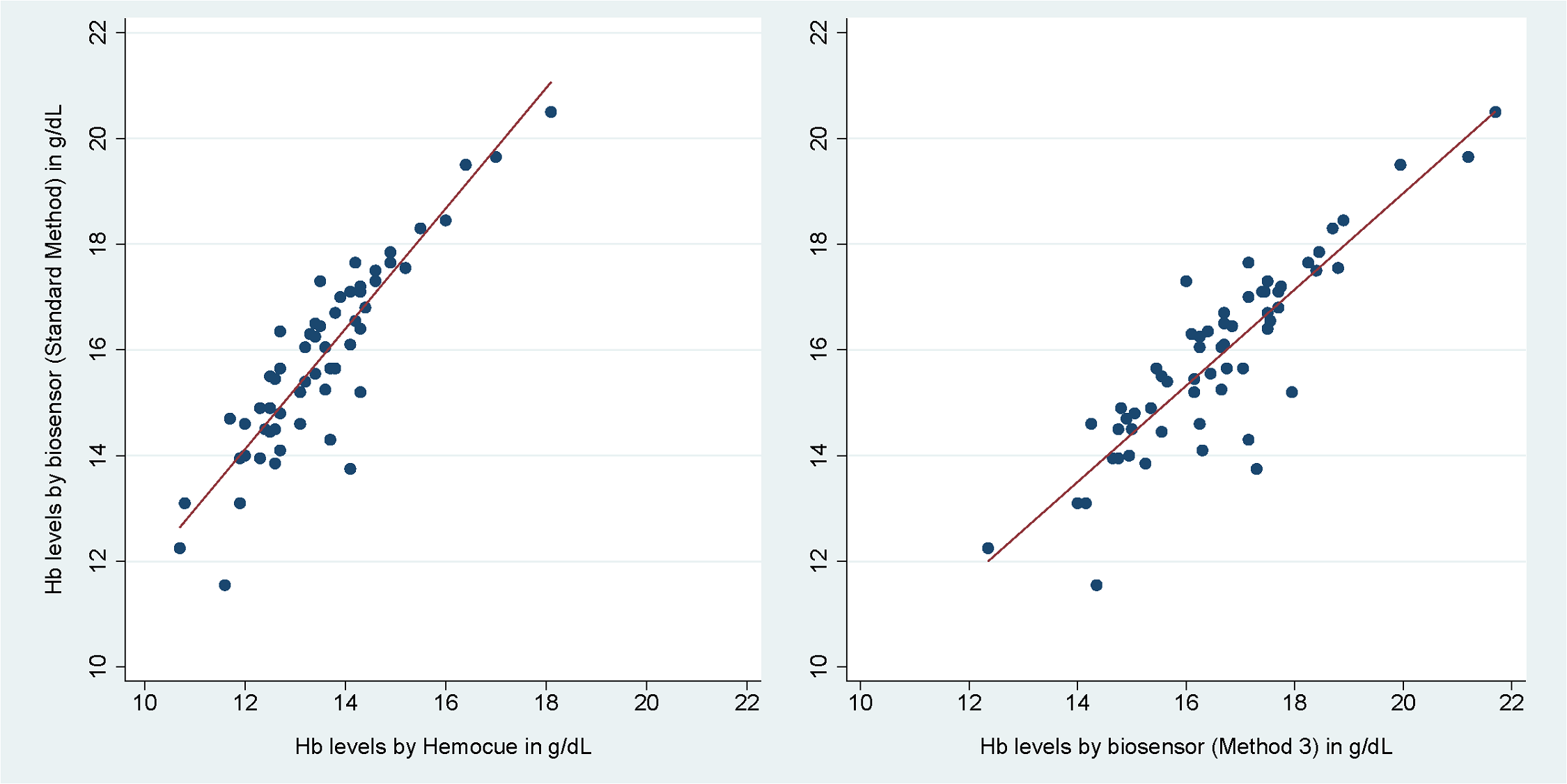

Supplement: S4 Fig — Scatterplot of Hb readings by the Biosensor using the Standard Method vs Hemocue (left, r = 0.895, p<0.001); and by the Biosensor using the Standard Method vs Method 3 (right, r = 0.881, p<0.001). (TIF) [file pone.0296708.s004.tif]

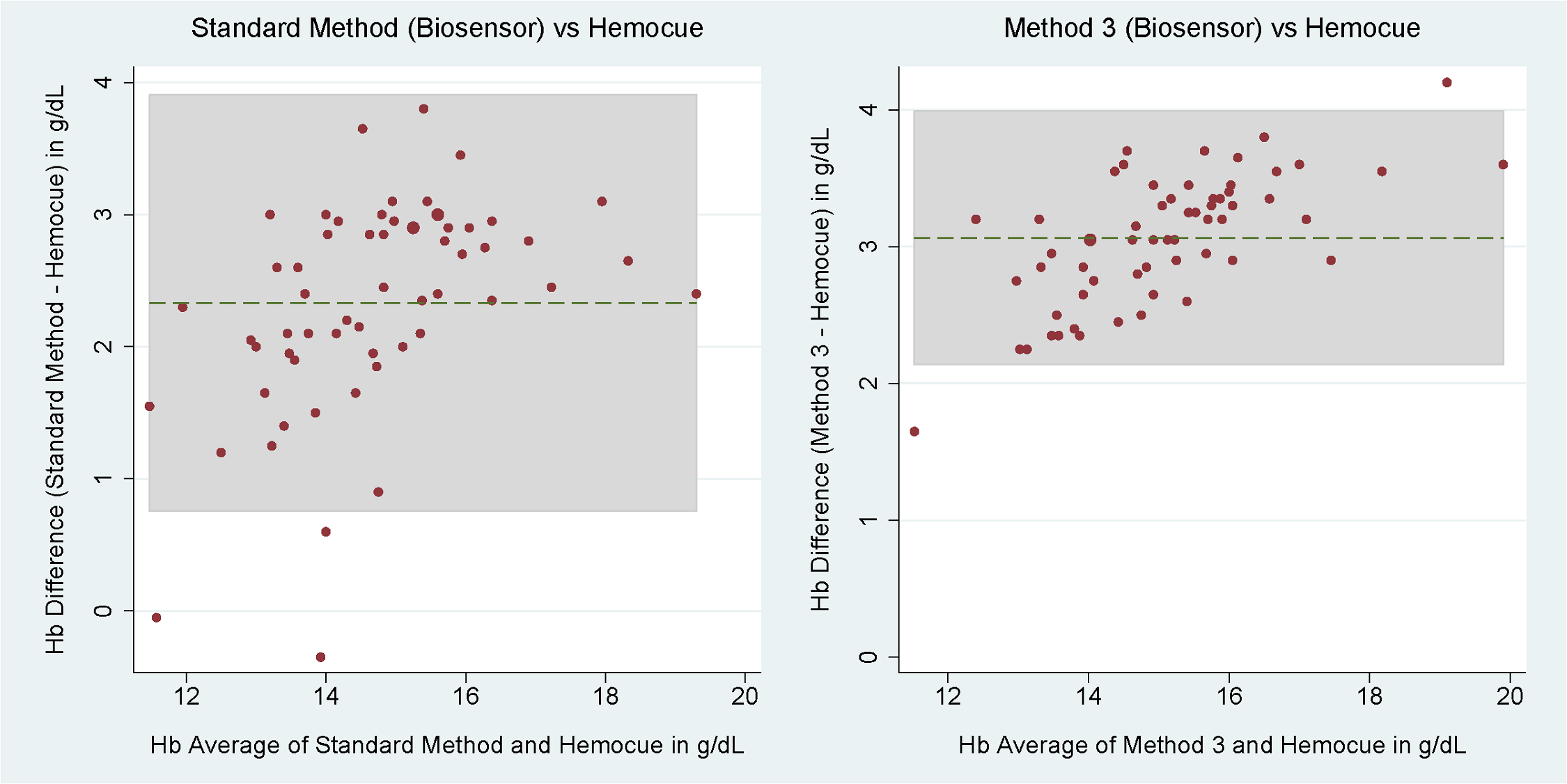

Supplement: S5 Fig — Bland-Altman plots comparing Hb readings by the Biosensor Standard Method (left) and Method 3 (right), both against Hemocue; the green dashed line marks the mean difference and the areas shaded in grey depicts the 95% limits of agreement. (TIF) [file pone.0296708.s005.tif]
