# Supplementary material for: Field assessment of the operating procedures of a semi-quantitative G6PD Biosensor to improve repeatability of routine testing
Source: PLoS One. 2024 Jan 19;19(1):e0296708. doi: 10.1371/journal.pone.0296708 (PMC10798449; doi:10.1371/journal.pone.0296708)
Supplement: S1 Table — Definition of a group: the absolute differences of all paired measurements taken using the same method at one site. (DOCX) [file pone.0296708.s006.docx]

**Table S1.** Summary of groupings and comparisons to assess the repeatability of G6PD measurements by Biosensor. Definition of a group: the absolute differences of all paired measurements taken using the same method at one site.

| **Element** | **Groups** | **Intergroup Comparisons** | **Intersite Comparisons** |
| --- | --- | --- | --- |
| **Pilot** | - Standard Method - Method 1 - Method 2 - Method 3 - Method 4 | Comparison of the median absolute differences of all methods: Kruskal-Wallis test | N/A |
| **Indonesia** | - Standard Method (Indonesia) - Method 3 (Indonesia) | Standard Method vs Method 3: Wilcoxon signed-rank test | - Indonesia vs Nepal (Standard Method): Mann-Whitney U test - Indonesia vs Nepal (Method 3): Mann-Whitney U test |
| **Nepal** | - Standard Method (Nepal) - Method 3 (Nepal) | Standard Method vs Method 3: Mann-Whitney U test |  |
