# Supplementary material for: Field assessment of the operating procedures of a semi-quantitative G6PD Biosensor to improve repeatability of routine testing
Source: PLoS One. 2024 Jan 19;19(1):e0296708. doi: 10.1371/journal.pone.0296708 (PMC10798449; doi:10.1371/journal.pone.0296708)
Supplement: S2 Table — (DOCX) [file pone.0296708.s007.docx]

**Table S2.** Summary of Hb, AMM, and G6PD status of Indonesian study population based on reference spectrophotometry.

|  | **Indonesia** |
| --- | --- |
| Median Hb in g/dL (IQR) ^*^ | 13.5 (12.6 to 14.3) |
| AMM (IQR) † | 11.14 (9.7 to 12.3) |
| **G6PD Deficient (Activity <30% AMM)** | |
| Male (%) | 0 |
| Female (%) | 1 (2.2%) |
| **G6PD Intermediate (Activity 30-70% AMM)** | |
| Male (%) | 1 (6.7%) |
| Female (%) | 1 (2.2%) |
| **G6PD Normal (Activity >70% AMM)** | |
| Male (%) | 14 (93.3%) |
| Female (%) | 43 (95.6%) |

*Based on measurements by Hemocue Hb 301

†Based on the G6PD activities of 41 males measured at the Indonesian field site (only 15 are participants of this study)
