# Supplementary material for: Field assessment of the operating procedures of a semi-quantitative G6PD Biosensor to improve repeatability of routine testing
Source: PLoS One. 2024 Jan 19;19(1):e0296708. doi: 10.1371/journal.pone.0296708 (PMC10798449; doi:10.1371/journal.pone.0296708)
Supplement: S3 Table — (DOCX) [file pone.0296708.s008.docx]

**Table S2.** Comparison of the median absolute difference between paired Biosensor Hb readings between methods and sites.

|  | **Median absolute difference between paired measurements (IQR) in g/dL** | |  |
| --- | --- | --- | --- |
|  | **Standard Method** | **Method 3** | **p-value (Standard Method vs Method 3)** |
| **Indonesia** | 0.6 (0.2-1.2) | 0.5 (0.3-0.9) | 0.262 |
| **Nepal** | 0.8 (0.3-1.3) | 0.3 (0.2-0.9) | 0.006 |
